# Supplementary material for: MetaRibo-Seq measures translation in microbiomes
Source: Nat Commun. 2020 Jun 29;11:3268. doi: 10.1038/s41467-020-17081-z (PMC7324362; doi:10.1038/s41467-020-17081-z)
Supplement: Supplementary file 10 — Supplementary Data 7 [file 41467_2020_17081_MOESM10_ESM.zip › File2/Confidence_High_Taxonomy/297648_out.krona.html]

Javascript must be enabled to view this page.

members
magnitude
magnitudeUnassigned
count
unassigned
taxon
rank

297648\_out

2

2
superkingdom
2

1
32066
phylum

class
203490
1

order
203491
1

1
1129771
family

genus
32067
1


SRS043018\_contig\_number\_13088
1
species
712357

phylum
976
1

1
200643
class

order
171549
1

815
family
1

genus
816
1


SRS971275\_contig\_number\_contig-100\_814.815
1
species
47678
